# Evaluation of disinfectants to prevent mechanical transmission of viruses and a viroid in greenhouse tomato production

**DOI:** 10.1186/s12985-014-0237-5

**Published:** 2015-01-27

**Authors:** Rugang Li, Fulya Baysal-Gurel, Zaid Abdo, Sally A Miller, Kai-Shu Ling

**Affiliations:** USDA-Agricultural Research Service, U.S. Vegetable Laboratory, 2700 Savannah Highway, Charleston, SC 29414 USA; Department of Plant Pathology, The Ohio State University, OARDC, 1680 Madison Avenue, Wooster, OH 44691 USA; USDA-ARS, South Atlantic Area, 950 College Station Road, Athens, GA 30605 USA

**Keywords:** *Pepino mosaic virus*, *Tomato mosaic virus*, *Tobacco mosaic virus*, *Potato spindle tuber viroid*, Disinfectant, Mechanical transmission, Greenhouse tomato

## Abstract

**Background:**

In recent years, a number of serious disease outbreaks caused by viruses and viroids on greenhouse tomatoes in North America have resulted in significant economic losses to growers. The objectives of this study were to evaluate the effectiveness of commercial disinfectants against mechanical transmission of these pathogens, and to select disinfectants with broad spectrum reactivity to control general virus and viroid diseases in greenhouse tomato production.

**Methods:**

A total of 16 disinfectants were evaluated against *Pepino mosaic virus* (PepMV), *Potato spindle tuber viroid* (PSTVd), *Tomato mosaic virus* (ToMV), and *Tobacco mosaic virus* (TMV). The efficacy of each disinfectant to deactivate the pathogen’s infectivity was evaluated in replicate experiments from at least three independent experiments. Any infectivity that remained in the treated solutions was assessed through bioassays on susceptible tomato plants through mechanical inoculation using inocula that had been exposed with the individual disinfectant for three short time periods (0–10 sec, 30 sec and 60 sec). A positive infection on the inoculated plant was determined through symptom observation and confirmed with enzyme-linked immunosorbent assay (PepMV, ToMV, and TMV) and real-time reverse transcription-PCR (PSTVd). Experimental data were analyzed using Logistic regression and the Bayesian methodology.

**Results:**

Statistical analyses using logistic regression and the Bayesian methodology indicated that two disinfectants (2% Virkon S and 10% Clorox regular bleach) were the most effective to prevent transmission of PepMV, PSTVd, ToMV, and TMV from mechanical inoculation. Lysol all-purpose cleaner (50%) and nonfat dry milk (20%) were also effective against ToMV and TMV, but with only partial effects for PepMV and PSTVd.

**Conclusion:**

With the broad spectrum efficacy against three common viruses and a viroid, several disinfectants, including 2% Virkon S, 10% Clorox regular bleach and 20% nonfat dry milk, are recommend to greenhouse facilities for consideration to prevent general virus and viroid infection on tomato plants.

## Background

Tomato (*Solanum lycopersicum* L.) is one of the world’s most economically important vegetables. According to the FAO statistics [1], a total of 162 million tons of tomatoes were produced worldwide in 2012. The top tomato producing countries are China, India, USA, and Turkey. Nearly 40% of fresh tomatoes sold in the U.S. supermarkets are produced in greenhouses [2]. Intensive crop manipulations in greenhouse tomato productions, such as grafting, bumblebee pollination, intercropping and deleafing, could lead to disease outbreaks from a number of mechanical transmitted viruses and viroids [3,4].

In the last decade, one of the most widespread viruses in greenhouse tomato productions in Europe and North America has been *Pepino mosaic virus* (PepMV), in the genus *Potexvirus* and family *Alphaflexiviridae* [5,6]. PepMV was discovered on pepino (*Solanum muricatum*) from Peru in 1980 [7]. It was first reported to infect greenhouse tomatoes in the Netherlands in 2000 [8] and has become endemic in Europe [9–15], Americas [16–21], and beyond [22–24].

In recent years, viroid disease outbreaks on tomatoes have been reported frequently both in Europe [25,26] and North America [4,27–31]. These viroids include *Potato spindle tuber viroid* (PSTVd), *Tomato chlorotic dwarf viroid* (TCDVd), *Mexican papita viroid* (MPVd), *Citrus exocortis viroid* (CEVd)*, Columnea latent viroid* (CLVd)*,* and *Tomato apical stunt viroid* (TASVd). Symptoms incited by these pospiviroids on tomatoes include plant stunting, leaf chlorosis and necrosis, with smaller size fruits to no fruit at all.

In addition to the endemic PepMV and the emerging pospiviroids, two tobamoviruses in the family *Virgaviridae*, *Tomato mosaic virus* (ToMV) and *Tobacco mosaic virus* (TMV), are frequently observed in greenhouse tomatoes [3].

Grafting is a common practice in greenhouse tomato seedling production. Contaminated tomato seeds used as scion and/or rootstock for grafting could also be an important source of initial virus inoculum [20,32]. Once a virus is established inside a greenhouse, rapid spread of the disease could occur due to many hands-on activities in greenhouse tomato production, and the nature of mechanical transmission of these concerned viruses and viroids [4,6]. Therefore, efficient and effective sanitation and disinfection protocols should be implemented to prevent or minimize the spread of these viruses and viroids in greenhouse tomato productions.

In recent years, several studies conducted to identify disinfectants that reduce the infectivity of plant viruses and viroids have been reported for ornamental plants [33–35], cucurbits [36], and greenhouse tomatoes [37,38]. Several common disinfectants (i.e., bleach and nonfat dry milk) have been shown to be effective in preventing the spread of certain viruses. However, currently there is still no clear understanding as to the effectiveness of disinfectants against a range of viruses and viroids encountered in greenhouse tomato productions. In this study, we investigated the efficacy of 16 disinfectants against three viruses (TMV, ToMV, and PepMV) and one viroid (PSTVd) commonly identified in greenhouse tomato productions. This work is to identify the most effective disinfectant(s) to prevent infection by these viruses and viroid.

## Results and discussion

### PepMV infection with cut- and rub-inoculation

In experiments to assess the virus infectivity remained in the treated solutions, two different inoculation methods were applied, cut- and rub-inoculation. In the first two cut-inoculation experiments for PepMV, since expression of typical disease symptoms (mosaic) from PepMV-infected tomato plants was not obviously visible, ELISA was used to determine the PepMV infection. Preliminary data indicated that there was a significant delay in the onset of PepMV infection from the cut-inoculated plants because extra time was needed to regenerate side shoots on the test plants after the primary shoot was removed by cut-inoculation. It took approximately 5 weeks post inoculation for PepMV to be detectable by ELISA achieving a 100% infection rate in plants from the positive control. In comparison, it typically took approximately only 1–3 weeks for PepMV to be detectable by ELISA in rub-inoculated plants. Such delay in the onset of PepMV infection to test plants through cut-inoculation hindered evaluation efficiency. In addition, the first two cut-inoculation experiments generated a higher infection rate (>50%) on test plants from all disinfectant solutions (Table 1).

**Table 1:** **Effectiveness of disinfectant solutions to deactivate pathogen infectivity as measured through experiments on tomato plants against** ***Pepino mosaic virus*** **(PepMV),** ***Potato spindle tuber viroid*** **(PSTVd),** ***Tomato mosaic virus*** **(ToMV), and** ***Tobacco mosaic virus*** **(TMV) infection**

**Disinfectant** ^**a**^	**PepMV** ^**b**^			**PSTVd** ^**c**^			**ToMV** ^**c**^			**TMV** ^**c**^		
	**0-10 sec**	**30 sec**	**60 sec**	**0-10 sec**	**30 sec**	**60 sec**	**0-10 sec**	**30 sec**	**60 sec**	**0-10 sec**	**30 sec**	**60 sec**
POS	3 3 3 3	3 3 3 3	3 3 3 3	2 2 3	2 2 3	2 2 3	3 3 3	3 3 3	3 3 3	3 3 3	3 3 3	3 3 3
GG	2 3 3 -	3 3 3 -	3 3 3 -	1 2 2	0 1 2	1 1 3	2 3 2	3 3 3	3 3 3	3 3 3	3 3 3	3 3 3
BS	2 3 3 -	3 3 3 -	3 2 3 -	1 2 2	1 2 3	0 1 3	3 3 3	3 3 3	3 3 3	3 3 3	3 3 3	3 3 3
GS	3 2 3 -	3 3 3 -	3 1 3 -	0 0 1	0 1 2	1 0 2	2 1 3	3 1 3	3 1 3	3 2 3	3 3 3	3 3 3
VOR	1 3 3 -	2 3 3 -	2 3 3 -	1 3 -	0 3 -	2 0 -	3 3 3	3 3 3	3 3 3	3 2 3	3 3 3	3 3 3
SD	3 3 3 -	3 2 3 -	3 2 3 -	0 3 3	1 2 3	1 3 1	3 2 3	3 3 3	3 3 2	3 2 3	3 1 3	3 3 3
DOG-0.1	2 3 3 -	3 3 3 -	3 2 3 -	2 2 -	2 3 -	1 2 -	2 3 3	3 3 3	3 3 3	3 3 3	3 3 3	3 3 3
DOG-0.2	2 1 3 -	3 2 3 -	3 1 3 -	1 3 -	1 3 -	3 2 -	3 3 3	3 3 3	3 3 3	3 3 3	3 3 3	3 3 3
KG	2 3 3 -	2 3 3 -	1 2 3 -	1 2 -	1 1 -	1 2 -	3 3 3	3 3 3	3 3 3	3 3 3	3 3 3	3 2 3
MF	2 1 3 2	0 3 2 3	2 2 3 1	0 2 -	2 1 -	1 1 -	0 0 1	0 1 2	0 2 3	2 2 3	3 2 3	2 2 2
MTF	0 2 3 1	0 2 3 1	2 2 2 1	0 0 3	0 0 1	1 0 0	1 0 2	0 0 2	0 0 2	0 1 1	0 0 2	0 0 3
SO	2 3 3 3	1 1 2 3	0 2 3 3	0 0 3	0 1 0	1 1 1	1 3 3	2 3 3	2 3 3	3 3 3	3 3 3	3 3 3
OCT	0 2 3 1	0 2 1 0	0 0 1 0	0 0 3	0 0 1	0 0 1	1 3 3	2 3 3	3 3 3	3 2 3	3 3 3	3 3 3
VS-0.5	0 3 1 -	0 3 2 -	0 3 2 -	1 2 -	2 1 -	3 2 -	3 2 1	3 3 3	3 3 2	3 2 3	3 1 3	3 3 3
TSP	0 2 0 2	0 2 2 3	0 2 0 0	2 2 -	2 3 -	0 3 -	2 2 1	0 2 0	2 2 0	2 0 2	2 0 2	3 0 2
LYSOL	3 3 2 0	3 3 0 0	3 3 0 0	0 0 1	0 0 2	0 1 0	0 0 0	0 0 0	0 0 0	0 0 0	0 0 0	0 0 1
**CLOROX**	**1 3 2 0**	**3 2 0 0**	**1 2 1 0**	**0 0 0**	**0 0 0**	**0 0 0**	**0 0 0**	**0 0 0**	**0 0 0**	**0 0 0**	**0 0 0**	**0 0 0**
**NFDM**	**0 3 0 0**	**0 3 1 0**	**1 3 1 0**	**0 0 1**	**0 0 0**	**2 0 0**	**1 0 0**	**0 0 0**	**0 0 0**	**0 0 0**	**0 0 0**	**0 0 0**
VS-1.0	1 3 0 0	2 2 0 0	2 1 0 0	2 1 0	2 1 0	2 2 0	2 0 0	0 0 1	0 0 0	3 1 0	3 0 2	1 0 3
**VS-2.0**	**0 3 0 0**	**2 3 0 0**	**1 3 0 0**	**0 0 0**	**0 0 0**	**0 0 0**	**0 0 0**	**0 0 0**	**0 0 0**	**0 0 0**	**0 0 0**	**0 0 0**
NEG	0 0 0 0	0 0 0 0	0 0 0 0	0 0 0	0 0 0	0 0 0	0 0 0	0 0 0	0 0 0	0 0 0	0 0 0	0 0 0

^a^The designations and application rates for each disinfectant used for these experiments are: POS: Virus or viroid infection only; GG: Greenhouse Guardian (1.1 g/L); BS: BioSide (0.78 mL/L); GS: Green-Shield (5.20 mL/L); VOR: Vortexx (1.95 mL/L); SD: SaniDate (3.82 mL/L); DOG-0.1: DES-O-GERM (1.0 mL/L); DOG-0.2: DES-O-GERM (2.0 mL/L); KG: KleenGrow (4 mL/L); MF: Menno Florades (30 mL/L); MTF: Menno-Ter forte (10 mL/L); SO: StorOx (9.77 mL/L); OCT: Octave (7.81 mL/L); VS-0.5: Virkon S (5 g/L); TSP: Trisodium phosphate (100 mL saturated solution/L); LYSOL: Lysol all-purpose cleaner (500 ml/L); CLOROX: Clorox Regular-Bleach (100 mL/L); NFDM: Nonfat dry milk (200 g/L); VS-1.0: Virkon S (10 g/L); VS-2.0: Virkon S (20 g/L); NEG: Buffer only. Three most effective disinfectants (Clorox, NFDM and VS-2.0) were highlighted with bold letters.

^b^There were four independent experiments for *Pepino mosaic virus* (PepMV). First two experiments were by cut-inoculation and the last two experiments by rub-inoculation. The count number of infected plants from a total of 3 inoculated plants at each exposure time point (0–10 sec, 30 sec and 60 sec) for each disinfectant is presented. “–” Represents no data available, as that particular disinfectant was not included in that experiment.

^c^ There were three replicated experiments for *Potato spindle tuber viroid* (PSTVd), *Tomato mosaic virus* (ToMV), and *Tobacco mosaic virus* (TMV). At each experiment, three tomato seedlings were inoculated with the designated inoculum at each exposure time point (0–10 sec, 30 sec, and 60 sec). Number of plants infected out of the total three inoculated at each time point are shown at each column in the table. Infected plants were determined through symptom observation followed by ELISA for PepMV, ToMV, and TMV and real-time RT-PCR for PSTVd. The count number of infected plants from a total of 3 inoculated plants at each exposure time point (0–10 sec, 30 sec and 60 sec) for each disinfectant is presented. “–” Represents no data available, as that particular disinfectant was not included in that experiment.

**Figure 1:**
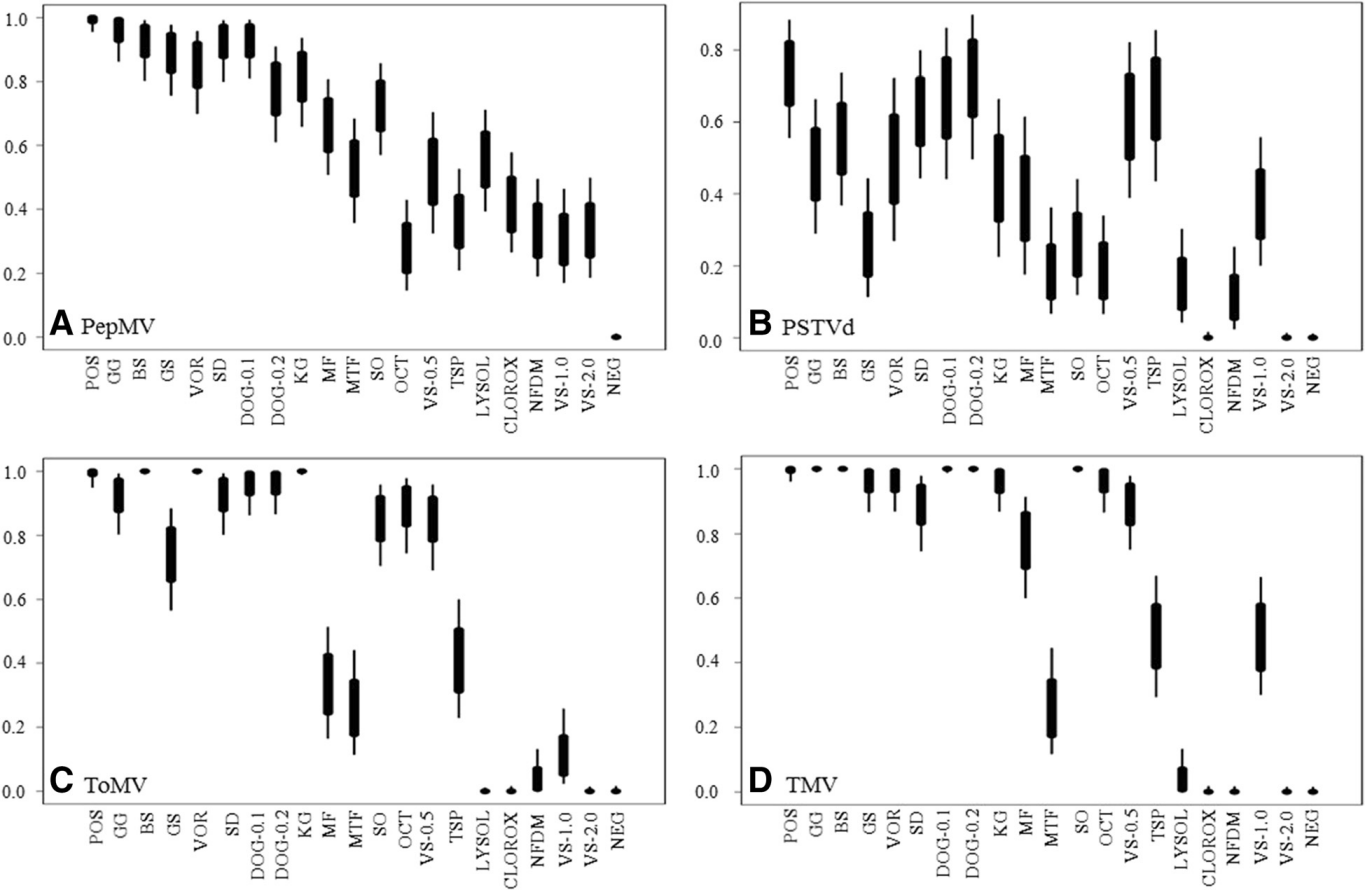
**Assessing the effectiveness of various disinfectants against virus infection.** Statistical analysis using the Bayesian method was used to evaluate the effect of different disinfectants against infectivity through mechanical inoculation on tomato plants of four viral and viroid pathogens, including **A)**. *Pepino mosaic virus* (PepMV), **B)**. *Potato spindle tuber viroid* (PSTVd), **C)**. *Tomato mosaic virus* (ToMV), and **D)**. *Tobacco mosaic virus* (TMV). In Bayesian analysis with a credibility interval (CI) of 1 representing 100% infection rate, meaning there was no effect in that treatment, whereas CI of 0 representing zero infection rate, meaning a full protection of treated plants by that disinfectant. The lower the CI value, the better effect that disinfectant against that pathogen. The higher the CI value, more plants were infected, thus the efficacy of that disinfectant was lower. Variable effects of each disinfectant are represented with a range of CI values generated from different replications. Those disinfectants with CI values not in overlap indicates significant differences between them. POS: positive control with pathogen only (PepMV, PSTVd, ToMV and TMV) without treatment; GG: Greenhouse Guardian (1.1 g/L); BS: BioSide (0.78 mL/L); GS: Green-Shield (5.20 mL/L); VOR: Vortexx (1.95 mL/L); SD: SaniDate (3.82 mL/L); DOG-0.1: DES-O-GERM (1 mL/L); DOG-0.2: DES-O-GERM (2 mL/L); KG: KleenGrow (4 mL/L); MF: Menno Florades (30 mL/L); MTF: Menno-Ter forte (10 mL/L); SO: StorOx (9.77 mL/L); OCT: Octave (7.81 mL/L); VS-0.5: Virkon S (5 g/L); TSP: Trisodium phosphate (100 mL saturated solution/L); LYSOL: Lysol all-purpose cleaner (500 ml/L); CLOROX: Clorox regular-bleach (100 mL/L); NFDM: Nonfat dry milk (200 g/L); VS-1.0: Virkon S (10 g/L); VS-2.0: Virkon S (20 g/L); NEG: negative control with inoculation buffer only.

Consequently, rub-inoculation method was introduced in the following two experiments for PepMV. Data from the third experiment for PepMV showed that Virkon S (1% and 2%) completely deactivated the PepMV infection. Only 1 or 2 in a total of 9 test plants tested positive from disinfectant solutions in nonfat dry milk, Trisodium phosphate, Lysol all-purpose cleaner, or Clorox regular bleach. The data from experiments 3 and 4 were similar (Table 1), with Virkon S (1% and 2%), Lysol all-purpose cleaner, nonfat dry milk, and Clorox regular bleach offering complete deactivation to PepMV infection. The data obtained from the first two cut-inoculation experiments were not very convincing due to a long delay for the onset of a positive infection, combined data analysis using pooled number of infected plants from four experiments by Bayesian analysis did not show significant difference among disinfectants (Figure 1A). However, if considering datasets only in the last two experiments by rub-inoculation, four disinfectants: Virkon S (1% and 2%), Lysol all-purpose cleaner (50%), nonfat dry milk (20%), and Clorox regular bleach (10%), were the most promising disinfectants against PepMV infection (Table 1).

### Efficacy of disinfectants against PSTVd

From a panel of 16 disinfectants used for PSTVd test, distinguishable effects to PSTVd infection were observed on the test tomato plants (Table 1). Due to consistent and high infection rates (30%) generated in the first two experiments, six disinfectants were excluded. The remaining 10 disinfectants that were used in the experiment 3 were Greenhouse Guardian, BioSide, Green-Shield, SaniDate, Menno-Ter Forte, StorOx, Octave, Lysol all-purpose cleaner, Clorox regular bleach, Nonfat dry milk, Virkon S (1% and 2%). After evaluation in three independent experiments, only two chemicals (10% Clorox regular bleach and 2% Virkon S) were able to completely deactivate PSTVd infectivity (Figure 1B, Table 1). Besides those two disinfectants with full effects, 20% nonfat dry milk and 50% Lysol also deactivated the PSTVd infectivity (Table 1). The Bayesian analysis supported such conclusions (Figure 1B).

### Efficacy of disinfectants against ToMV

ToMV infection on tomato seedlings of ‘VTV’ hybrid was so severe that a positive infection resulted in plant death. Mosaic and necrotic lesions to the upper leaves were observed within one week post inoculation. In two weeks post inoculation, symptom expression was so obvious that some severely infected plants began to die. Thus, scoring of plant infection was easily done through symptom observation. Data sets on the efficacy tests of 16 disinfectants against ToMV were consistent among three experiments (Table 1). The most effective disinfectants against ToMV were 10% Clorox regular bleach, 2% Virkon S, and 50% Lysol all-purpose cleaner (Table 1). Only a small proportion (<10%) of plants in treatments with 20% nonfat dry milk or 1% Virkon S resulted in infection (Table 1). Three other chemicals (3% Menno-Florades, 1% Menno-Ter Forte, and 10% Trisodium phosphate) prevented 30% plants from infection. The Bayesian statistical analysis supported above conclusions (Figure 1C).

### Efficacy of disinfectants against TMV

TMV infection on the tomato ‘VTV’ plants was also very severe without treatment, similar to those by ToMV, which resulted in plant death. The treatment effects were consistent in all 3 experiments (Table 1). Three disinfectants with full effects against TMV infection were 10% Clorox regular bleach, 2% Virkon S, and 20% nonfat dry milk. The 50% Lysol all-purpose cleaner was also effective in deactivating TMV infectivity, only 1 of 9 plants infected in one of the three experiments (Table 1). Another disinfectant, 1% Menno-Ter Forte, had partial effect which resulted in less than 9 of 27 test plants infected from three experiments. The results from Bayesian analysis also supported the above conclusion (Figure 1D).

### Impact of application rate on efficacy

**Figure 2:**
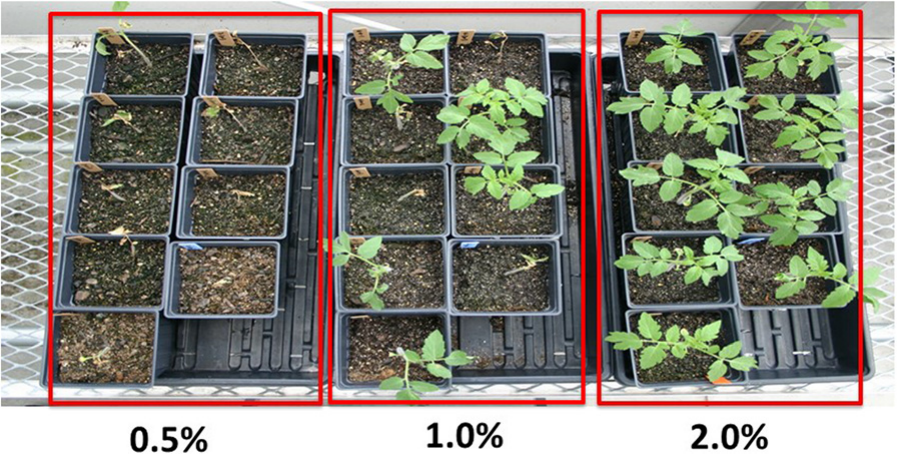
**Effects of various concentrations of Virkon S in deactivating** ***Tobacco mosaic virus*** **(TMV) infectivity as assessed in bioassays through rub-inoculation upon exposure for 30 sec using Virkon S at 0.5% (left) with no protection, at 1% (middle) with partial protection, and at 2% (right) with full protection.**

The effect of disinfection is also dependent on the application rate. When comparing Virkon S in three concentrations (0.5%, 1% and 2%), the impact of application rate to TMV infection were observed (Figure 2). In general, 0.5% Virkon S had little effect to slow down virus or viroid transmission (Table 1). Partial protection to TMV was observed when 1% Virkon S was used (Figure 2). However, when 2% Virkon S solution was used, full protection from TMV infection was observed (Figure 2). Taken together, 2% Virkon S achieved the most consistent effects against four tomato virus and viroid pathogens: PepMV, PSTVd, TMV, and ToMV (Table 1 and Figure 1).

### Efficacy of selected disinfectants upon storage

**Figure 3:**
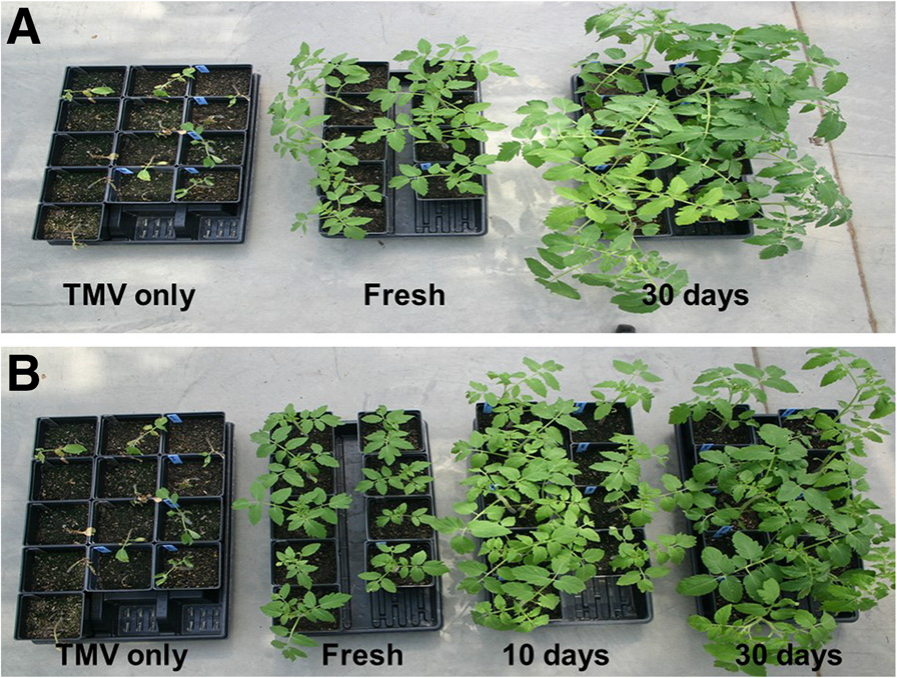
**Stability of prepared disinfectants in prolong storage for their effect in deactivating** ***Tobacco mosaic virus*** **(TMV) infectivity. A)**. Comparative effectiveness of freshly prepared 10% Clorox® bleach solution (Fresh) and a similarly prepared solution in storage at room temperature (20–30°C) for 30 days. The positive control (TMV only) without treatment was used to assess the TMV infectivity in the inoculum. **B)**. Comparative effectiveness of 2% Virkon S solutions from a freshly prepared (Fresh), those in storage for 10 days (10 days) and 30 days (30 days) after preparation at room temperature (20–30°C). A positive control (TMV only) was used to assess TMV infectivity in the inoculum.

To address concerns over the stability of disinfectants in prolong storage, the two most effective disinfectants (10% Clorox regular bleach and 2% Virkon S) were examined against TMV infection. Each treatment was performed through mixing an equal volume of TMV inoculum with a 2X stock solution of a respective disinfectant. In the case of Clorox regular bleach, the 2X stock solution (20% Clorox regular bleach) was stored at room temperature (20-30°C) for 30 days. Virkon S, 4% stock solution (2X) was prepared and stored for two time periods, 10 days and 30 days. At each storage time point, treatments were carried out by mixing an equal volume of a prepared virus inoculum with a 2X stock solution for 30 seconds just before used for a bioassay. Results showed that in comparison to that of a freshly prepared solution, the ability to deactivate TMV infectivity of Clorox regular bleach in storage for over 30 days was equally effective (Figure 3A). Virkon S, stock solutions (2X) in storage for 10 days or 30 days were equally effective to deactivate TMV infectivity in comparison to those of a freshly prepared solution. While all the test plants in the positive control without treatment were severely infected and dying, plants treated with a freshly prepared solution or those solutions in storage for 10 days and 30 days were fully protected from TMV infection (Figure 3B).

A total of 16 disinfectants were evaluated for their effectiveness in preventing transmission of three major tomato viruses (PepMV, ToMV, and TMV) and one viroid (PSTVd) through mechanical inoculation on tomato seedlings. This study demonstrated consistently the broad spectrum effects from two common disinfectants (2% Virkon S and 10% Clorox regular bleach) in deactivating the infectivity of PepMV, PSTVd, ToMV, and TMV (Table 1). Four other disinfectants (1% Virkon S, 50% Lysol all-purpose cleaner, 20% nonfat dry milk, and 1% Menno-Ter Forte) also had promising efficacy in deactivating certain target pathogens (Table 1, Figure 1).

The three exposure times (0–10 sec, 30 sec, or 60 sec) in treatment of inocula by disinfectants were selected to mimic the short time intervals in cutting tissues between plants during tomato crop work in a greenhouse, such as grafting, deleafing, or fruit harvesting. However, there were no significant differential responses observed among the three time intervals. These results suggested that the effects of those effective disinfectants against virus and viroid infectivity were immediate upon mixing and exposure. With such swift action from the effective disinfectants, there is no need to wait after dipping for a disinfectant to achieve its full function.

Growers are concerned about how long a diluted disinfectant could be stored in a greenhouse without losing its efficacy. Our tests demonstrated that there was no major reduction in the effect of a disinfectant in deactivating TMV infectivity when those ready-to-use solutions of Virkon S and Clorox regular bleach were stored for more than 30 days. It is not surprising given the long shelf life of these highly stable products. However, caution should be considered when using disinfectants with high amount of organic matter, such as a large quantity of leaf sap, which could compromise the disinfectant activity [37].

Results obtained in screening of 16 disinfectants against three viruses in tomato were consistent with some conclusions achieved in previous studies from other plant-virus systems. Hu et al. (33) found that undiluted skim milk inactivated *Cymbidium mosaic virus* (CyMV) on a local lesion host and commercial bleach inactivated both CyMV and *Odontoglossum ringspot virus* (ORSV) at 10% or 20% concentration. Kamenova and Adkins [34] reported that in experiments mimicking plant propagation and pruning, 10% bleach solution and 20% nonfat dry milk completely prevented *Hibiscus latent Fort Pierce virus* infection in hibiscus. In searching for effective disinfectants in ornamental production to control TMV transmission during propagation, Lewandowski et al. [35] discovered that several common disinfectants including 20% nonfat dry milk or 10% household bleach completely eliminated TMV transmission to petunias. They also showed that treatment of contaminated tools with 1% Virkon S solution or 20% nonfat dry milk (7% protein) also significantly reduced the incidence of TMV-infected petunias. Coutts et al. [36] examined 13 disinfectants for their effectiveness in inactivating ZYMV in cucurbits. They discovered that none of test plants became infected when nonfat dry milk (20% w/v) or bleach solution (42 g/L NaOCl, diluted 1:4) was used. Matsuura et al. [37] found that active component NaOCl at a concentration of 0.5% (10% bleach solution) or more was the most effective in disinfecting *Tomato chlorotic dwarf viroid* (TCDVd)-contaminated scalpels. Wintermantel [38] demonstrated that 0.5% sodium hypochlorite treatment for two seconds was sufficient for inactivation of potyviruses during pruning operations and superior to quaternary ammonium solution. Although the virus-plant systems tested were different, the general effective role of three disinfectants, 1% or 2% Virkon S, 10% Clorox regular bleach (containing 0.525% NaOCl), and nonfat dry milk (containing 7% protein), were consistently able to deactivate virus infection. This is the first time in this study to show that nonfat dry milk can also suppress the viroid infectivity. The possible mode of action for nonfat dry milk against viroid infectivity is awaiting further study. Taken together, it is reasonable to believe that these disinfectants could be generally applied to other virus/viroid-plant systems. In addition, Virkon S and Clorox regular bleach have been shown to be effective against some common bacterial and fungal pathogens on tomato [39].

## Conclusions

With their broad spectrum effects against infection on treated tomato plants for three viruses and a viroid, 2% Virkon S, 10% Clorox regular bleach and 20% nonfat dry milk could be recommended to greenhouse vegetable growers to protect plants from virus infection in various crop work activities, including grafting, deleafing, and fruit harvesting. A suitable disinfectant for greenhouse tomato production should satisfy several criteria, including: short contact time, broad efficacy against viruses and viroids or even other plant pathogens, safe for workers, not corrosive to infrastructure, not phytotoxic to plants, and economic. Although Virkon S is relatively expensive and also corrosive, it is the most promising disinfectant and has already been proven effective against human and animal viral pathogens [40,41]. Clorox regular bleach is widely used at home for killing common household germs; however, the strong corrosive effect to the greenhouse structure and tools and the potential phytotoxic effect on tomato plants might not be welcomed by greenhouse tomato growers. Nonfat dry milk, the third most efficient disinfectant, is safe for application, economic to use, and seems to satisfy the above disinfectant criteria. By rotating the application of several effective disinfectants with different mode of actions, the risk of a virus and viroid outbreak on greenhouse tomatoes may be brought under control.

## Materials and methods

### Sources of viruses and viroid and their maintenance

The PepMV isolate (TX10-01) was isolated from a diseased tomato plant collected in Texas in 2010 and determined by sequence analysis to be genotype CH2 [6]. ToMV (isolate V13-07) and TMV (isolate U1) were provided by Heinz Co. and Monsanto, respectively. PSTVd (isolate NC12-01) was isolated from North Carolina in 2012 [4]. Active cultures of each individual virus and viroid were maintained through rub-inoculation on tomato cultivars ‘Rutgers’ or ‘Moneymaker’ and kept separately inside an insect-proof bug-dome in a greenhouse. Those symptomatic tomato leaves from plants inoculated 4–8 weeks prior and confirmed to be infected by a particular virus or viroid were collected as inoculum sources to assess the efficacy of disinfection.

### Plant preparation and growth

Certified healthy tomato seeds (cv. VTV, F_1_ hybrid indeterminate Saladette tomato) were provided by Monsanto. Experiments were conducted in a greenhouse (temperature of 25-30°C, a natural sun-light period of 14 hours). For each replicated experiment (one virus or a viroid treated with 16 disinfectants and controls), over 220 seeds were individually sowed in 5-inch pots filled with soil-less potting mix (Sunshine mix, SunGro Horticulture) in a greenhouse. The test plants were maintained in each individual pot on trays to keep them separated in space to prevent potential cross contamination. Normal plant maintenance activities, including daily watering, weekly fertilizing and occasional spraying to control insects, were performed.

### Disinfectants

A total of 16 products were selected for evaluation in this study, their active ingredients and application rates are listed in Table 2. Product concentrations were determined based either on the labeled rates, earlier studies by others with different plant-virus systems [33–38], or from grower experiences. Each product with 2× stock of application rate was prepared within 1 hour before use in the same day.

**Table 2:** **List of disinfectants and their application rates used to evaluate their efficacy as disinfectants against three tomato viruses and one viroid**

**No.**	**Disinfectant**	**Application rate**	**Designation**	**Active ingredients**	**Manufacturer**
1	Clorox regular bleach	10% (100 mL/L)	CLOROX	5.25% Sodium hypochlorite (NaOCl)	The Clorox Company. Oakland, CA, USA
2	KleenGrow	0.4% (4 mL/L)	KG	7.5% Didecyl dimethyl ammonium chloride	Pace Chemicals. Burnaby, BC, Canada
3	Virkon S	0.5% (5 g/L)	VS-0.5	20.4% Potassium peroxymonosulfate, 1.5% Sodium chloride	DuPont Chemical Solutions, Wilmington, DE, USA
	Virkon S	1.0% (10 g/L)	VS-1.0		
	Virkon S	2.0% (20 g/L)	VS-2.0		
4	Greenhouse Guardian	0.11% (1.1 g/L)	GG	Trichloromelamine	GermFreePlanet, Tucson, AZ, USA
5	Green-Shield	0.52% (5.20 mL/L)	GS	10% n-alkyl [60% C14, 30% C16, 5% C12, 5% C18] Dimethyl benzyl ammonium chloride, 10% n- alkyl [68% C12, 32% C14] Dimethyl ethylbenzyl ammonium chloride	BASF, Research Triangle Park, NC, USA
6	Non-fat dry milk (Sanalac)	20% (200 g/L)	NFDM	34.78% Protein	ConAgra Food, Omaha, NE, USA
7	Vortexx	0.195% (1.95 mL/L)	VOR	6.9% Hydrogen peroxide, 4.4% Peroxyacetic acid, 3.3% Octanoic acid	Ecolab Center, St. Paul, MN, USA
8	Octave	0.781% (7.81 mL/L)	OCT	7.52% Hydrogen peroxide, 0.94% Peroxyoctanoic acid, 2.72% Octanoic acid	Ecolab Center, St. Paul, MN, USA
9	BioSide	0.078% (0.78 mL/L)	BS	15% Peroxyacetic acid, 22% Hydrogen peroxide	Enviro Tech Chemical Services, Modesto, CA, USA
10	SaniDate	0.382% (3.82 mL/L)	SD	23.0% Hydrogen peroxide, 5.3% Peroxyacetic acid	Biosafe Systems, Glastonbury, CT, USA
11	StorOx	0.977% (9.77 mL/L)	SO	27% Hydrogen dioxide	Biosafe Systems, Glastonbury, CT, USA
12	Lysol all-purpose cleaner	50% (500 mL/L)	LYSOL	0.1% alkyl [50% C14, 40% C12, 10% C16] Dimethylbenzyl ammonium saccharinate	Reckitt Benckiser, Parsippany, NJ, USA
13	DES-O-GERM	0.1% (1 mL/L)	DOG-0.1	Poly hexamethylenebiguanide hydrochloride, Benzalkonium chloride	Des-O-Germ (PTy), Africa-Australia-Mauritius-New Zealand
	DES-O-GERM	0.2% (2 mL/L)	DOG-0.2		
14	Menno Florades	1% (10 mL/L)	MF	9% (w/v) Benzoic acid	Menno Chemie-Vertrieb Gmbh, Norderstedt, Germany
15	Menno-Ter forte	1% (10 mL/L)	MTF	32.5% Didecyl dimethyl ammonium chloride	Menno Chemie-Vertrieb Gmbh, Norderstedt, Germany
16	Trisodium phosphate	10% (100 mL/L saturation)	TSP	Trisodium phosphate	Fisher Scientific, Fair Lawn, NJ, USA

### Preparation of virus inoculum

The virus and viroid sources were maintained on tomato plants in individual insect-proof domes in a greenhouse. Virus inoculum was prepared by grinding symptomatic tomato leaves (1:5 w/v) in a plastic bag containing saline phosphate buffer, pH 7.0 (140 mM NaCl, 8 mM Na_2_HPO_4_.12H_2_0, 1.5 mM KH_2_PO_4_, 2.7 mM KCl and 0.8 mM Na_2_SO_3_) using a Homex-6 tissue homogenizer (BioReba AG, Switzerland). PSTVd inoculum was prepared using the above listed saline phosphate buffer or 1× TE buffer (10 mM Tris–HCl and 1 mM EDTA, pH 8.0). The freshly prepared virus or viroid inoculum was maintained on ice prior to use.

### Virus inoculation

The processed virus/viroid inoculum was kept on ice in a 50-mL gamma irradiation-sterilized conical crew cap centrifuge tube (USA Scientific) prior to use. Equal volumes (0.3 ml) of the prepared disinfectant stock solution and virus/viroid inoculum were mixed in a 1.5 mL Eppendorf tube. Each disinfectant and inoculation combination was left at room temperature for 0–10 sec, 30 sec or 60 sec prior to use for inoculation.

For PepMV only, the first 2 experiments were done using cut-inoculation, which simulated to the deleafing process. The cut-inoculation was done by dipping a clean surgical blade to the treated solutions as stated above and cut off a leaf or a shoot on 3 tomato seedlings at each exposure time intervals (0–10 sec, 30 sec and 60 sec).

Rub-inoculation was conducted by rubbing gently with a cotton-swab (Q-tip) soaked in the treated solution onto 3 tomato seedlings lightly dusted with Carborundum (320-grit, Fisher Scientific) at 2–3 leaf stage. Each treated inoculum was inoculated on 3 tomato seedlings for each exposure time period (0–10 sec, 30 sec or 60 sec). In each experiment, 3 plants at each time point were inoculated with the same virus or viroid inoculum that were mixed with an equal volume of water as positive controls. Same number of mock-inoculated plants with the inoculation buffer were served as negative controls. The entire process from inoculum preparation to the completion of an inoculation experiment took 2–3 hours. As a control, the infectivity of a virus inoculum that remained in the solution at the end of each experiment was tested by inoculating 3 additional tomato seedlings to confirm symptom expression. The virus/disinfectant solution was washed off gently with tap water from the inoculated plants after application.

Tests on PSTVd, ToMV and TMV, consisted of 3 repeat experiments and were done using the rub-inoculation method. For PepMV, 2 experiments were done with the cut-inoculation method and 2 experiments were done with the rub-inoculation method. The data from two inoculation methods were compared for PepMV. The inoculated plants were randomized on a bench and maintained in a greenhouse for 4–6 weeks post inoculation for symptom observation. ELISA for virus detection and qRT-PCR for PSTVd were performed as described in the following to confirm each virus or viroid infection. The experimental plant materials were bagged, autoclaved, and disposed of after completion of each experiment. For each pathogen, the experiment to each disinfectant was repeated for 3 times. Due to the limitation in greenhouse space and to prevent potential cross contamination, only plants inoculated by the same type of pathogen at each time were placed in the same greenhouse. The entire experiments were carried out from April 2012 to June 2013 in an environmental controlled greenhouse.

### Efficacy of selected disinfectants in storage

To assess the efficacy of selected disinfectants in storage, 2X stock solutions of Clorox regular bleach (20%) were stored at room temperature (25°C) for 30 days and then used to treat the TMV inoculum with an exposure time for 30 sec. In comparison, a freshly prepared 20% Clorox regular bleach was used as a control and a non-treated TMV inoculum was used to assess the virus infectivity in the inoculum. For Virkon S, the 2X stock solution (4%) was assessed after storage for 10 to 30 days, in comparison with a freshly prepared stock solution to treat the TMV inoculum with a 30 sec exposure. A non-treated TMV inoculum was included as a positive control. Preparation and maintenance of plants for inoculation and the method on rub-inoculation were described as above.

### Enzyme-linked immunosorbent assay (ELISA)

To assess virus infection on the test plants for PepMV, TMV, and ToMV, a standard ELISA method was used following the manufacturer’s instructions (Agdia, USA) with minor modifications. For sample processing, a small leaf tissue (ca. 150 mg) was collected from individual inoculated plant in a plastic bag. After addition of 2.0 ml 1X tissue extraction buffer (General) (BioReba, Switzerland) into each bag, the tissue was grounded thoroughly with a Homex-6 homogenizer (BioReba, Switzerland). The ELISA plate was pre-coated with 100 μl of primary antibody for PepMV (catalog no. 13001, Agdia, USA), ToMV (catalog no. 35400, Agdia, USA) or TMV (catalog no 57400, Agdia, USA) in suitable dilution in the coating buffer overnight at 4°C. After washing with 1x PBS-Tween buffer for 3–4 times, the above prepared leaf crude extract (100 μl) was added to the wells of the antibody-coated plate and incubated for 2 h at 37°C or overnight at 4°C. A positive control (virus-infected), negative control (not virus-infected), and non-template control (extraction buffer only) were included in each plate. After rigorous washing, the plate was filled with 100 μl of secondary antibody-Alkaline phosphatase enzyme conjugate (1:200 dilution in the conjugate buffer) and incubated for 2 h at 37°C. Color development was generated by incubation with 100 μl of p-nitrophenyl phosphate (PNP) substrate and absorbent readings were measured at 405 nm with an ELISA plate reader (SpectraMAX PLUS 384, Molecular Devices, USA). Absorbance values at least twice over that of the negative control were considered positive.

### Real-time RT-PCR

Real-time RT-PCR reaction was used to determine the infection of PSTVd and carried out using a Takara One Step Ex Taq qRT-PCR kit (Clontech Laboratories, USA) on an Mx3000P qPCR machine (Stratagene/Agilent Technologies, USA). RNA template was prepared with a simple dilution of crude tissue extract method [42]. Approximately 150 mg leaf tissue was ground in a plastic bag filled with 1.5 ml of 0.1 M Tris–HCl buffer (pH 8.0). The crude extract was diluted 1:100 with diethylpyrocarbonate (DEPC)-treated water and 0.5 μl of diluted extract was added to the reaction mixture. The primers for PSTVd (PSTV-231 F: 5’-GCCCCCTTTGCGCTGT-3’; PSTV-296R: 5’-AAGCGGTTCTCGGGAGCTT-3’) and TaqMan probe (PSTV-251 T: CY5-CAGTTGTTTCCACCGGGTAGTAGCCGA- BHQ2) were derived from published sequences [43] and synthesized by IDT (Coralville, IA, USA). Each real-time RT-PCR reaction mixture (10 μl) consisted of 5 μl of 2X reaction buffer, 0.25 μl Ex Taq HS mix (5 U/μl), 0.25 μl RTase mix (5 U/μl) in Takara’s Premix EX Taq (Clontech, USA), 0.25 μl forward primer (20 μM), 0.25 μl reverse primer (20 μM), and 0.25 μl TaqMan probe (10 μM), 0.19 μl of ROX reference dye (500X dilution, Clontech, USA), and 0.5 μl RNA. The thermocycling program included an initial cycle for reverse transcription at 50°C for 30 min and a denaturation at 95°C for 2 min, then 40 cycles of 95°C for 10 sec and 55°C for 30 sec. A positive control (virus-infected), negative control (non- virus-infected), and non-template control were included in each test. A cycle threshold value (Ct) above 31.00 at a threshold fluorescence level of 0.025 was determined as a background reaction.

### Statistics analysis

To determine whether there was any significant effect between disinfectants on each target pathogen, the number of plants infected for each disinfectant solution and exposure time were pooled and analyzed using Logistic regression and the Bayesian methodology [44]. To do so, a Markov Chain Monte Carlo (MCMC) approach was used as implanted in JAGS [45] through the R statistical software [46]. The credibility intervals (CI) were designated between 0–1, where those CI values between disinfectant solutions with no overlap are considered significantly different. If the CI value in a disinfectant solution was equal to zero, that result indicated complete deactivation of the pathogen infectivity was achieved and none of the test plants was infected. If a CI value approached 1, the result indicated there was no effect of that disinfectant against the pathogen infectivity, as all the test plants were infected.

## Abbreviations

PepMV: *Pepino mosaic virus*
PSTVd: *Potato spindle tuber viroid*
ToMV: *Tomato mosaic virus*
TMV: *Tobacco mosaic virus*
ELISA: Enzyme linked immunosorbent assay
qRT-PCR: Quantitative (real-time) reverse transcription polymerase chain reaction
